# Supplementary material for: The interactome of intact mitochondria by cross-linking mass spectrometry provides evidence for coexisting respiratory supercomplexes
Source: Mol Cell Proteomics. 2017 Dec 8;17(2):216–32. doi: 10.1074/mcp.RA117.000470 (PMC5795388; doi:10.1074/mcp.RA117.000470)
Supplement: Supplemental Data [file supp_RA117.000470_133922_0_supp_23978_fzffwf.zip › spectra_annotation/mito_spectra_annotation/52-1-10-1-19-1.pdf]

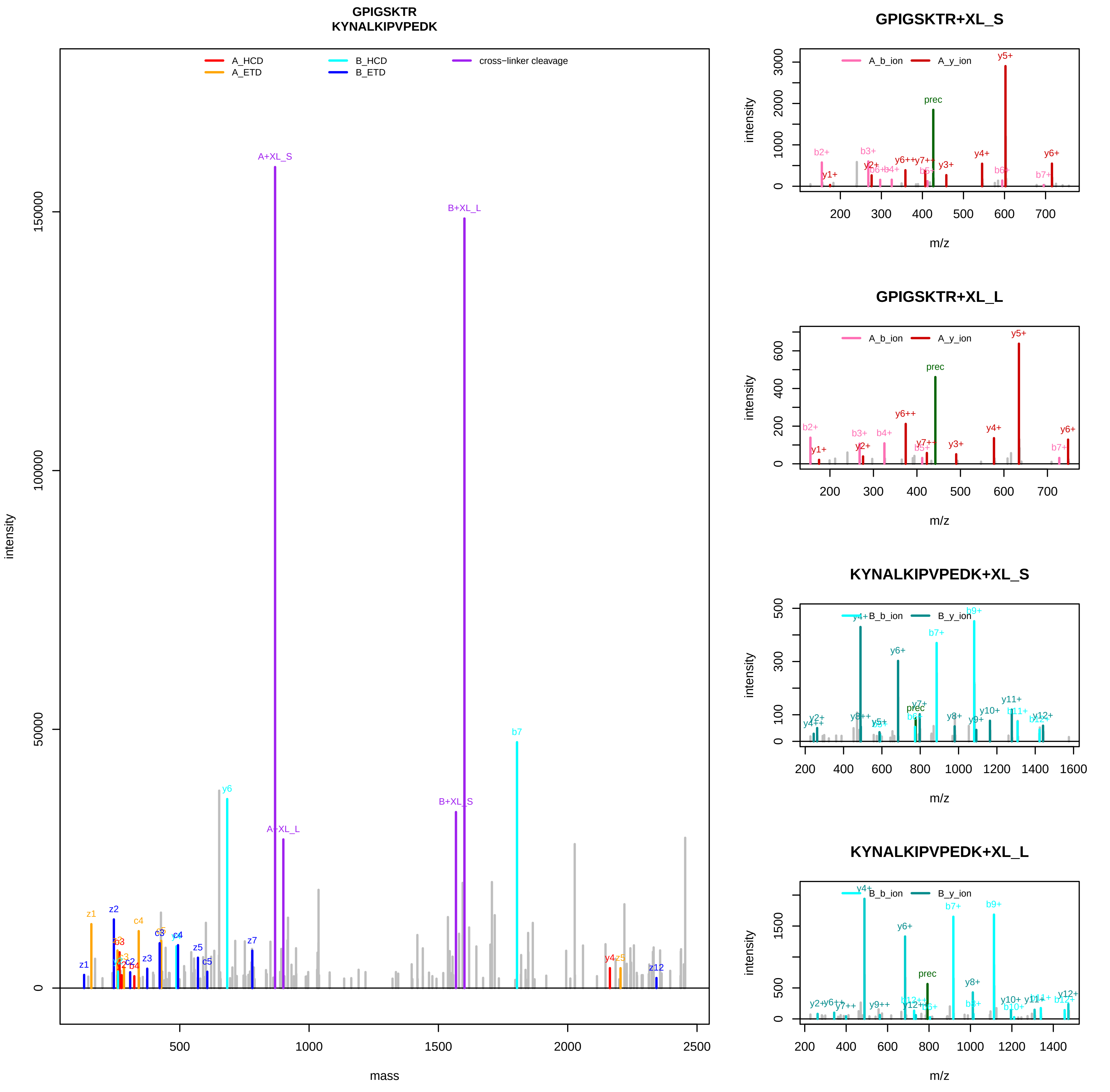
GPIGSKTR
KYNALKIPVPEDK
A_HCD
A_ETD
B_HCD
B_ETD
cross−linker cleavage
A+XL_S
B+XL_L
B+XL_S
A+XL_L
y6
b7
z1
z2
c4
c3
c4
z5
c5
z7
z3
y4
z5
z12
intensity
mass
0
50000
100000
150000
500
1000
1500
2000
2500
GPIGSKTR+XL_S
A_b_ion
A_y_ion
prec
y5+
y4+
y6+
y3+
b2+
b3+
y1+
b7+
GPIGSKTR+XL_L
KYNALKIPVPEDK+XL_S
B_b_ion
B_y_ion
b9+
b7+
y6+
y4+
y11+
y10+
KYNALKIPVPEDK+XL_L
y8+
b9+
b7+
y6+
y4+
y12+
m/z
